# Evolutionary characterization and pathogenicity of a porcine G9P[23] rotavirus with gene segments linked to canine and giant panda strains

**DOI:** 10.1016/j.virusres.2025.199600

**Published:** 2025-06-15

**Authors:** Xi Li, Jingjing Wang, Yuankui Zhang, Yarong Zhao, Wenjun Liu, Yanli Shi

**Affiliations:** a Beijing Biomedicine Technology Center, Zhaofeng Hua Biotechnology (Nanjing) Co., LTD, Beijing, China; b CAS Key Laboratory of Pathogenic Microbiology and Immunology, Institute of Microbiology, Chinese Academy of Sciences (CAS), Beijing, China

**Keywords:** Porcine rotavirus, Isolation, Whole-genome sequencing, Genome analysis, Pathogenicity

## Abstract

Porcine rotavirus A (RVA) has emerged as an increasingly consequential zoonotic pathogen, causing severe intestinal disorders across diverse mammalian species, including humans. During of an outbreak that struck nursing piglets with diarrhea, a porcine G9P[23] rotavirus, named as RVA/Pig-wt/China/ZJ03/2022/G9P[23] (hereafter referred to as ZJ03), was identified. To further elucidate the evolutionary diversity of ZJ03, a comprehensive analysis of all genome segments was conducted. The genome constellation was identified as G9-P[23]-I5-R1-C1-M1-A8-N1-T1-E1-H1. Nucleotide sequence identity and phylogenetic analyses indicated that the VP3 and NSP1 genes of ZJ03 are most closely related to the corresponding genes of the giant panda strain and the dog strain, respectively, showing the highest homology at 95.73 % identity and 94.64 %. The remaining genes demonstrated the most intimate relationship with porcine strains. Their highest homology levels ranged from 95.98 % to 99.49 % similarity. Therefore, evidence suggests interspecies transmission and genetic reassortment events between porcine, canine, and giant panda rotavirus strains. To evaluate the pathogenicity of ZJ03 strain, we experimentally infected 3-day-old piglets oral inoculation with the PoRV ZJ03 strain at a dose of 2 × 10^5.5 TCID_50_/ml per piglet. The infection resulted in severe diarrhea in all piglets, which occurred at 48 h post-infection (hpi), accompanied by sustained viral shedding and characteristic small intestinal villous atrophy, indicating significant damage to the intestinal epithelium. In vitro, ZJ03 exhibited efficient replication kinetics in MA104 cells, reaching peak titers of 10^9.25 TCID_50_/mL at 36 h post-infection. This study reports the first documented case of a novel porcine G9P[23] rotavirus with gene segments linked to canine and giant panda strains in mainland China, characterized by high viral titer and virulence. The findings highlight the emergence of a previously unrecorded RVA strain with significant virological and ecological implications.

## Highlights

• First report of a porcine G9P[23] rotavirus with gene segments linked to canine and giant panda strains in mainland China.
• The genome analysis of the ZJ03 strain reveals that the VP3 gene (which exhibits the highest homology to the VP3 genome segment of the giant panda) and the NSP1 gene (which exhibits the highest homology to the NSP1 genome segment of the dog) carry a risk of cross-species transmission.
• Experimental infection induces severe diarrhea and intestinal damage in piglets within 48 h.
• ZJ03 strain exhibits high replication efficiency (peak titer 10^9.25 TCID50/mL) in MA104 cells.
• Highlights zoonotic risks and ecological implications for wildlife conservation, particularly giant pandas.

## 1 Introduction

Rotaviruses (RVs) are a leading cause of severe gastroenteritis in both animals and human infants worldwide. The first porcine rotavirus strain was isolated from neonatal pigs by Woode and Bridge in 1974 (Zhang et al., 2024), marking a pivotal advancement in understanding the etiology of swine diarrhea. Neonatal and suckling piglets exhibit high susceptibility to RV infection, with morbidity rates exceeding 90 % in commercial swine populations, resulting substantial economic losses for the global livestock industry (Midgley et al., 2012; Vlasova et al., 2017). Beyond its impact on animal health, RVs pose a grave public health challenge. Rotavirus infections are associated with over 200,000 fatalities annually among children under the age of five, predominantly in Southern Asia and nations within sub-Saharan Africa (Ghonaim et al., 2025). Porcine rotavirus infection manifests clinically as severe watery diarrhea, vomiting, anorexia, and dehydration in swine, particularly in neonatal piglets (Gao et al., 2023; Patelet al., 2013). Despite interspecies transmission barriers and host range restrictions, genomic reassortment events between human and animal rotavirus A strains (RVAs) have been documented, driving its genetic diversity (Akari et al., 2023). This evolutionary plasticity has profound implications for both medical and veterinary epidemiology, as emerging evidence suggests porcine RVA strains may serve as zoonotic reservoirs for human, canine, and other animal infections (Qiao et al., 2024). The potential for cross-species spillover highlights the need for integrated surveillance strategies to mitigate the global health burden of rotavirus disease, which remains a leading cause of pediatric gastroenteritis and livestock productivity losses worldwide.

Rotaviruses (RVs), members of the *Sedoreoviridae* family, possess an 11-segmented double-stranded RNA genome encoding six structural proteins (VP1-4, VP6-7) and six non-structural proteins (NSP1-6) (Crawford et al., 2017; Matthijnssens et al., 2022). Currently, ten distinct species of rotavirus are classified, designated from A to J (LeClair et al., 2023), with RVA being of greatest biomedical significance due to its broad host range encompassing humans, swine, and diverse mammalian species (Vlasova et al., 2017; Gomez-Rial et al., 2020). The standardized genotype nomenclature (Gx-P[x]-Ix-Rx-Cx-Mx-Ax-Nx-Tx-Ex-Hx) reflects the genetic diversity across all 11 genomic segments, each encoding a distinct viral protein (Chen et al., 2019). Notably, G9 has emerged as the predominant RVA genotype in southern China between 2021-2023, accounting for 56.55 % of sequenced samples, followed by G5, G3, and G4, as well as other genotypes (Zhang et al., 2024; Qiao et al., 2024). This regional prevalence pattern corroborates global observations identifying swine as key reservoirs for human G9 RVAs, with documented interspecies reassortment events between porcine and human strains (Wu et al., 2017; Miao et al., 2022). Despite its clinical significance across veterinary and public health sectors, the G9P[23] genotype remains underrepresented in swine population studies, particularly regarding its evolutionary origins, host adaptation mechanisms, and zoonotic transmission dynamics. Systematic investigations into these knowledge gaps are imperative to inform targeted surveillance and intervention strategies.

## 2 Materials and methods

### 2.1 Ethics statement

The animal experiments were carried out in accordance with the Chinese Laboratory Animal Regulations, the Guidelines for the Care of Laboratory Animals (Ministry of Science and Technology of the People's Republic of China), and Laboratory Animal Requirements for Environment and Housing Facilities (GB 14925-2010; national Technical Committee for Standardization). This research protocol was approved by the Laboratory Animal Ethical Committee of Kemu Feng Biotechnology (Beijing) Co., LTD under license number KMF20230620-2.

### 2.2 Sampling and virus isolation

In 2022, fecal samples were collected from piglets with acute gastroenteritis at a pig farm in Zhejiang Province. Initially, the samples were subjected to reverse transcription-polymerase chain reaction (RT-PCR) using specific primers targeting PoRV, Porcine epidemic diarrhea virus (PEDV), Porcine deltacoronavirus (PDCoV), and Transmissible gastroenteritis virus (TGEV) to screen for the presence of RVA (primers summarized in Table 1). Following RT-PCR, the PoRV-positive samples were processed by mixing 1 g of each fecal sample with 1 mL of PBS, centrifuging at 8,000 rpm, and the supernatants were filtered through 0.22 μm membranes. The filtered liquid was treated with trypsin (20 μg/mL, 37 °C, 30 min), and subsequently, MA104 cells were inoculated. MA104 cells were cultured in DMEM+10 % FBS, and the confluent monolayer was washed three times with PBS. Then, 1 mL of filtrate was inoculated into each T25 cell culture flask and incubated for 2 h. Post-adsorption, cells were maintained in DMEM + 5 μg/mL trypsin at 37 °C with 5 % CO₂ and monitored for cytopathic effects. After six passages and three plaque purifications, the virus strain was further passaged three times to obtain the stable isolate ZJ03 (F9).

### 2.3 Sequence determination and analysis

The RNA extracted from the F9 generation of strain ZJ03 was submitted to Beijing Qingke Biotechnology Co., Ltd. for next-generation sequencing (NGS) analysis. The Illumina Novaseq 6000 platform was employed for this purpose. Subsequently, the NGS results were uploaded to the National Center for Biotechnology Information (NCBI) for BLAST comparison. Genotypes were determined following the method described by Matthijnssens et al. According to this proposed porcine rotavirus (PoRV) genotyping method, a genotype was considered consistent when the percentage of nucleotide sequences in 11 genome fragments exceeded a specific threshold. To further explore the genetic origin of ZJ03, phylogenetic trees were constructed using the neighbor - joining method in MEGA7 software.

### 2.4 Pathogenicity experiments

In this study, eight 3-day-old piglets were procured from a family farm with no observable signs of diarrhea. RT-PCR testing confirmed that all piglets were negative for PoRV, PEDV, TGEV and PDCoV (Table 1). Sera samples from the sows exhibited no neutralizing antibodies against PoRV. The piglets were randomly allocated into two groups: an experimental group comprising five individuals and a control group of three. They were housed separately in different rooms. During the entire experiment, the piglets were fed milk powder every three hours. The experimental group challenged with PoRV was orally inoculated with the PoRV ZJ03 strain (2 ml/piglet, at a titer of 1.0 × 10^5.5 TCID_50_/ml), while the control group received an oral inoculation of DMEM (2 ml/piglet). Throughout the study, all piglets were monitored every 24 h for clinical signs, including diarrhea and virus shedding. Clinical mental state scores were assigned based on the following evaluation criteria: 0 = normal; 1=mild lethargy (slow movement, head down); 2=moderate lethargy (stands but often lies down); 3=severe lethargy (lies down, rarely stands); 4=extremely severe lethargy (recumbent, moribund). The occurrence of diarrhea was observed, and its severity was recorded according to a previous report: 0=normal; 1=soft feces (cow- pie consistency); 2=very soft, approaching liquid; 3=liquid with some solid content; 4=watery diarrhea with no solid content (Wang et al., 2018a). After the piglets were euthanized, intestinal tissues were collected for viral load analysis using reverse transcription-quantitative polymerase chain reaction (RT-qPCR) and pathological examination. The results were presented as the mean±standard deviation (SD) of three independent experiments, and data graphs were generated using GraphPad Prism 8.0 (GraphPad Software, USA).

**Table 1:** The detection primers of enterovirus.

Primers Name	Primer sequence	Product size (bp)
PoRV-VP7-F	ATGTATGGTATTGAATATACCACAGTT	785
PoRV-VP7-R	TGTATWAYWGCTACRTTYTCYCTTGGTCC	
PDCoV-M-F	GGCAAATTATTGTTTTCATTGCGATCATATGGGCGC	625
PDCoV-M-R	CTTATACAGGCGAGCGTCACCGGCCTTTGAAG	
TGEV-S-F	TCGCAATAATAGTAATGACCTTTAT	480
TGEV-S-R	TTAAACCACCAAAGGTCTACAA	
PEDV-M-F	GCATCCTTATGGCTTGCATCAC	337
PEDV-M-R	GTGCCAGATGAAGCATTGACTGAACG	

### 2.5 Reverse transcription quantitative polymerase chain reaction (RT-qPCR)

RT-qPCR amplification was carried out using the SYBR Green Realtime PCR Master Mix (Vazyme, China). Total RNA was extracted with the Viral RNA Extraction Kit (TIANGEN, China) and reverse - transcribed using the FastKing-RT SuperMix containing DNase (TIANGEN, China), strictly following the manufacturer's instructions. The generated cDNA was then used for downstream qPCR analysis. In brief, real-time RT-qPCR with the SYBR Green qPCR Master Mix was performed using the 7500 software, in accordance with the instructions provided in the detection kit. A specific primer pair targeting the VP6 gene (qPCR-VP6-F: TRTTTCCGCAAGCRCCRCCATT; qPCR-VP6-R: ACCHGGTGGAAATACYGGTCCT) was utilized to quantify the virus RNA levels in the samples. Quantification of viral RNA was achieved by using a standard curve derived from serial 10-fold dilutions of the VP6 plasmid. The viral RNA copies in intestinal tissue samples were converted to total viral RNA copies per gram of intestinal tissue. All samples were analyzed in triplicate.

### 2.6 Histology and immunohistochemistry (IHC)

To analyze the histopathological changes, each intestinal tissue sample was fixed in 10 % formalin, dehydrated in 70 % graded ethanol, and then embedded in paraffin for sectioning and mounting onto slides. The sections were stained with hematoxylin and eosin (HE) following the standard procedures provided by Shanxi Agricultural University. Pathological alterations in the tissue were examined by observing the stained slides. For the detection of PoRV-specific antigen on the fixed sections, a VP6-specific polyclonal antibody (diluted 1:2000) was used as the primary antibody. The secondary antibody was HRP-conjugated goat anti-rabbit IgG (zsbio, China) (diluted 1:200). Images were captured using an Olympus IX15 light microscope.

### 2.7 Electron microscopic observation

To visualize the PoRV virion particles, MA104 cells were harvested when cytopathic effect (CPE) was observed in over 80 % of the cells. The cell - free virus suspension was filtered through 0.45 μm filters and then centrifuged at 30,000 RPM at 4 °C for 1.5 h using an ultracentrifuge (Beckman) equipped with a TYPE 70Ti rotor to pellet the virus particles. The virus pellets were resuspended in 1 ml of PBS buffer and further purified by sucrose density gradient centrifugation (30 %, 45 %, and 60 % sucrose solutions). Subsequently, the purified viral samples were negatively stained with phosphotungstic acid and examined under an electron microscope.

### 2.8 Indirect immunofluorescence assay (IFA)

The trypsin-treated virus was inoculated into MA104 cells seeded in 6-well plates at a multiplicity of infection (MOI) of 0.01, with a blank control included. After 12 h of incubation, the culture medium was discarded, and the cells were fixed in cold anhydrous ethanol at 4 °C for 20 min. Subsequently, the cells were washed three times with PBS, each wash lasting five minutes. Next, the cells were blocked with 5 % skim milk powder at 37 °C for 1 h. Following the blocking step, the cells were washed three more times with PBS, each wash for five minutes. Then, the cells were incubated with rabbit polyclonal antibodies against the VP6 protein (prepared in our laboratory, diluted to a concentration of 1:2000) at 37 °C for 1 h. After being washed three times with PBS for five minutes each, an FITC-labeled goat anti-rabbit fluorescent secondary antibody (Proteintech, diluted to a concentration of 1:200) was added, and the incubation was continued at 37 °C for 1 h. Finally, the cells were washed three times with PBS for five minutes each and then treated by adding 200 μl of PBS. The samples were observed under an inverted fluorescence microscope.

### 2.9 Virus titration

The isolated virus stock was activated by supplementing with trypsin to a final concentration of 20 μg/ml, followed by incubation at 37 °C for 30 min. Serial 10-fold dilutions (10⁻¹ to 10⁻⁹) were prepared in serum-free DMEM maintenance medium containing 5 μg/ml trypsin. Confluent MA104 cell monolayers in 96-well plates were pre-washed three times with PBS prior to inoculation. Each dilution was inoculated into six replicate wells (0.1 mL/well), while six control wells received 0.1 mL of maintenance medium alone. Plates were incubated at 37 °C under 5 % CO₂ for 5–7 days. Cytopathic effects (CPE) were monitored daily, and TCID₅₀ values were calculated using the Reed-Muench method. Data from three independent experiments were expressed as mean ± SD, with graphical analyses performed using GraphPad Prism 8.0 (GraphPad Software, USA).

## 3 Results

### 3.1 Virus Isolation, identification, and growth curves of ZJ03

**Fig. 1:**
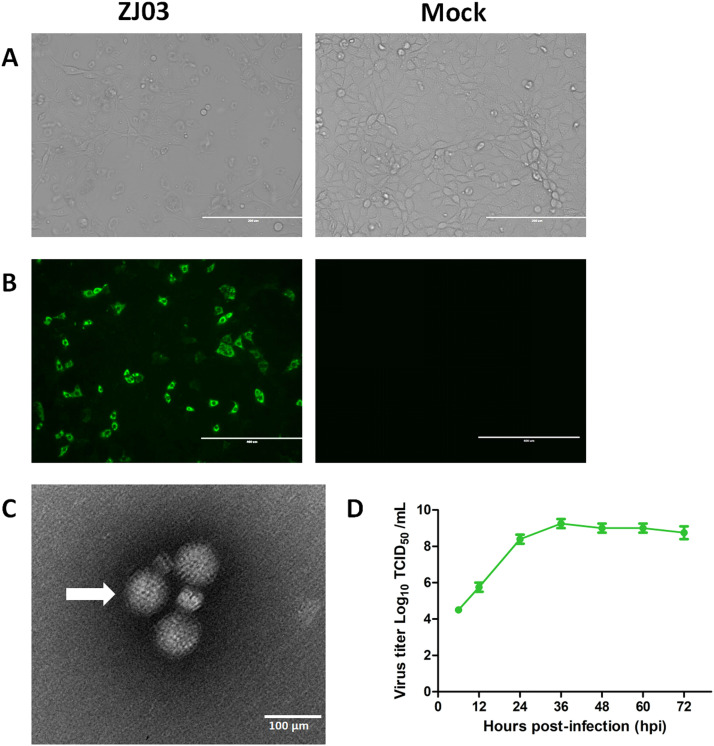
Virus Isolation, identification, and growth curves of ZJ03. (A) CPE of MA104 cells infected with ZJ03. (B) Fluorescence were stained for RVA-VP6 (Green). (C) Electron microscopic images of purified PoRV particles. (D) Growth kinetics of ZJ03 in MA104 cells. Data was presented as mean ± SD by triplicates.

We attempted to isolate PoRV from PCR-positive diarrhea samples that were free of PEDV, PDCoV, and TGEV. Fortunately, a PoRV, designated as ZJ03, was successfully isolated from the samples of a farm in Zhejiang Province, China. Upon initial inoculation onto MA104 cells, the ZJ03 isolate manifested pronounced cytopathic effects (CPE) characteristic of rotavirus (RV) infection. During the serial propagation of the virus, a typical CPE, characterized by enlarged, rounded cells and cellular debris, was observed at 24 hpi (Fig. 1A). Moreover, the propagation of PoRV strain ZJ03 in MA104 cells was verified through the detection of PoRV antigens via immunofluorescence assay (IFA) using polyclonal antibodies targeting the VP6 protein (Fig. 1B). As illustrated in Fig. 1B, PoRV-VP6 protein-specific immunofluorescence was detected in the majority of cells at 12 hpi, with the VP6 protein primarily localized in the cytoplasm. In contrast, no CPE or VP6 staining was observed in the mock. Purified ZJ03 virus particles harvested from infected MA104 cells were characterized using transmission electron microscopy (TEM). Negative-staining EM revealed a wheel-like morphology consistent with spherical virions, measuring approximately 70 nm in diameter (Fig. 1C). TEM analysis confirmed efficient replication of PoRV in the MA104 cell system. Viral growth kinetics were determined by quantifying 50 % tissue culture infective dose (TCID_50_) titers at serial time points post-infection. The ZJ03 strain exhibited a peak titer of 1 × 10^9.25 TCID_50_/mL, with a rapid exponential phase observed between 6 and 36 h post-infection (hpi) (Fig. 1D).

### 3.2 NGS results and the genome sequences of ZJ03

RNA was extracted from the F9 generation virus and sent to Beijing Qingke Biotechnology Co., Ltd. for next-generation sequencing (NGS) analysis. Unbiased high-throughput sequencing was performed using the Illumina NovaSeq 6000 platform, yielding a total of 8,122,322 reads with an average length of ∼150 bp for the viral genome. Complete coding sequences were successfully resolved for all 11 genome segments of the sequenced strain (ZJ03). The full-length genome of strain ZJ03 measured 17,940 base pairs (bp), with individual gene segments as follows: VP1 (2,474 bp), VP2 (2,751 bp), VP3 (2,621 bp), VP4 (2,403 bp), VP6 (1,484 bp), VP7 (1,144 bp), non-structural proteins NSP1 (1,153 bp), NSP2 (1,134 bp), NSP3 (1,199 bp), NSP4 (851 bp), and NSP5 (726 bp). Comprehensive sequence analysis, including multiple sequence alignment, nucleotide identity calculations, and BLASTn searches against the NCBI database, revealed that this strain belongs to the G9P[23] genotype with a genomic constellation of G9-P[23]-I5-R1-C1-M1-A8-N1-T1-E1-H1 (Table 2). BLASTn results indicated that most gene segments (VP1, VP2, VP4, VP6, VP7, NSP2, NSP3, NSP4, NSP5) shared the highest nucleotide sequence identities (95.98 %–99.49 %) with porcine rotavirus A (RVA) strains. Notably, the VP3 segment showed the highest homology (95.73 %) to a giant panda-derived RVA isolate, while the NSP1 segment exhibited significant similarity (94.64 %) to a canine RVA sequence. These findings suggest that strain ZJ03 may represent a novel reassortant virus derived from porcine, giant panda, and canine RVA ancestors, highlighting potential interspecies transmission and genetic recombination events.

**Table 2:** The highest nucleotide sequence indentities between ZJ03 and other known RVA strains.

Gene	Closely related strains	Homology	Genotype	Accession No.
VP7	RVA/Pig/CHN/2013/LY-2	96.24 %	G9	KT820778.1
VP4	RVA/Pig/CHN/2021/SX-2021	95.98 %	P[23]	OP650547.1
VP6	RVA/pig/CHN/2013/DZ-1	97.79 %	I5	KT820766.1
VP1	RVA/pig-tc/CHHeN/05E/2023/G9P[23]	96.88 %	R1	PP025945.1
VP2	RVA/Pig/CHN/BH/2023/G12P[7]	97.68 %	C1	PP391051.1
VP3	RVA/Giant panda/CHN/2008/CH-1	95.73 %	M1	HQ641295.1
NSP1	RVA/dog-tc/CHN/SCCD-A/2017/G9P[23]	94.64 %	A8	MH910069.1
NSP2	RVA/Pig-wt/VNM/2012/14226_42	98.68 %	N1	KX363417.1
NSP3	RVA/Pig/China/2022/YT	96.15 %	T1	OR232957.1
NSP4	RVA/Pig/China/2008/LLP48	97.20 %	E1	KJ126820.1
NSP5	RVA/Pig/CHN/2021/SD-1	99.49 %	H1	ON676179.1

### 3.3 Phylogenetic analysis of the 11 segments

**Fig. 2:**
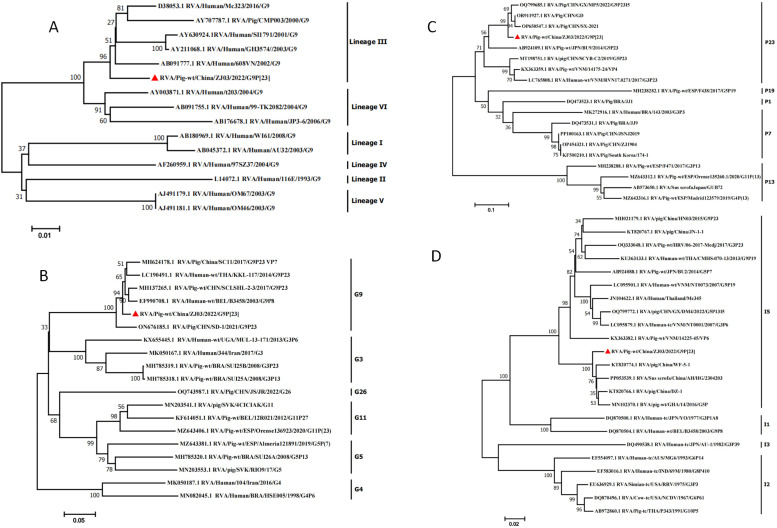
Phylogenetic analysis based on the nucleotide sequences of the VP7,VP4,VP6 segments from ZJ03 and other strains by MEGA 7 using the neighbor-joining (NJ) method and 1,000 bootstrap replicates. (A) Phylogenetic analysis revealed that ZJ03 strain can be classified into the lineage III. (B) Phylogenic trees based on VP7 gene from ZJ03. (C) Phylogenic trees based on VP4 gene from ZJ03. (D) Phylogenic trees based on VP6 gene from ZJ03.

Phylogenetic analysis of the VP7 gene segment confirmed the classification of the G9 genotype into six distinct lineages (Fig. 2A). Lineages I, II, IV, and V were exclusively associated with human rotavirus A (RVA) isolates, whereas lineages III and VI demonstrated zoonotic potential through their presence in both human and porcine hosts (Zhang et al., 2015). Sequence alignment analysis revealed that strain ZJ03 exhibited nucleotide sequence identities ranging from 94.64 % (lineage II) to 99.49 % (lineage III), with reference strains from different G9 lineages, corresponding to genetic distances of 0.51 %–5.36 %. Specifically, ZJ03 shared the highest identity (99.49 %) with the human RVA strain 608VN (GenBank accession no.: AB091777.1; lineage III) and the lowest identity (94.64 %) with the human strain 116E (GenBank accession no.: L14072.1; lineage II).

The phylogenetic analysis of the VP7 gene (Fig. 2B) demonstrated a close evolutionary relationship between strain ZJ03 and porcine G9 genotype rotavirus A (RVA) strains isolated in China, including SD-1 (GenBank accession no.: ON676185.1) and SC11 (GenBank accession no.: MH624178.1). These findings, combined with the geographic distribution of G9-positive isolates across multiple Chinese provinces (e.g., Shandong, Sichuan), suggest widespread circulation of this genotype in swine populations. Phylogenetic analysis of the VP4 gene of ZJ03 (Fig. 2C) grouped within the same clade with Chinese porcine P[23] RVA strains SX-2021 (GenBank accession no.: OP650547.1) and GD (GenBank accession no.: OR911927.1). Conversely, it diverged from other P[23] isolates including Vietnamese strain 14175_24 (GenBank accession no.: KX363359.1), Japanese strain BU9 (GenBank accession no.: AB924109.1), and Chinese strain SCYB-C2 (GenBank accession no.: MT198751.1). The VP6 gene of ZJ03 was classified as genotype I5 based on sequence identity 97.8 % to reference strain DZ-1 (GenBank accession no.: KT820766.1; Fig. 2D). Phylogenetic reconstruction further confirmed close genetic relatedness (97.2 % nucleotide identity) to another Chinese porcine RVA isolate, WF-5-1 (GenBank accession no.: KT820774.1), suggesting regional conservation of this genotype.

**Fig. 3:**
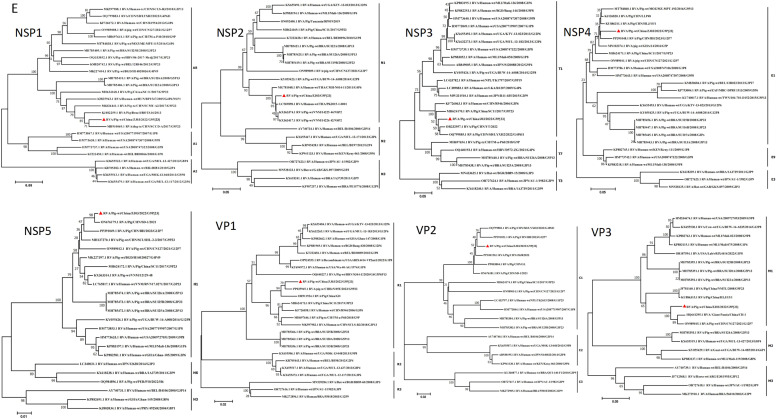
Phylogenetic analysis based on the nucleotide sequences of the NSP1, NSP2, NSP3, NSP4, NSP5, VP1, VP2 and VP3 segments from ZJ03 and other strains by MEGA 7 using the neighbor-joining (NJ) method and 1,000 bootstrap replicates. Phylogenic trees based on the nucleotide of NSP1, NSP2, NSP3, NSP4, NSP5, VP1, VP2 and VP3 genes from ZJ03.

BLAST analysis of the VP1-VP3 and NSP1-NSP5 genes of ZJ03 revealed genotype classifications of R1, C1, and M1 for VP1-VP3, and A8, N1, T1, E1, and H1 for NSP1-NSP5, respectively (Fig. 3). The VP3 segment of ZJ03 clustered with the VP3 gene of giant pandas and a porcine strain identified in China. The NSP1 segment of ZJ03 exhibited close phylogenetic affinity to a canine strain. Phylogenetic analysis suggests that ZJ03 likely represents a reassortment event involving rotavirus segments from pigs, giant pandas, and dogs, highlighting the potential for cross-species transmission.

### 3.4 Comparison of VP4 and VP7 antigenic epitopes of the ZJ03 strain

**Fig. 4:**
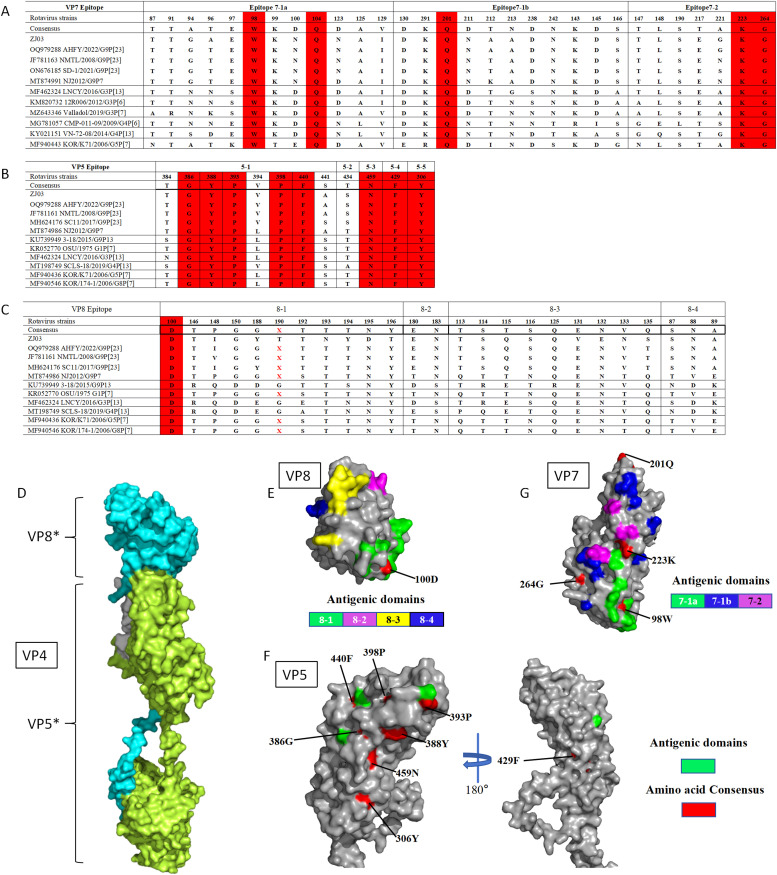
Comparison of VP4 (VP5* and VP8*) and VP7 antigenic epitopes in different strains. (A) Neutralizing epitopes on the VP7 protein. (B) Neutralizing epitopes on the VP5 protein. (C) Neutralizing epitopes on the VP8 protein. The red letters represent the mutilation of amino acids. (D) The structure of the VP4 protein. (E) The structure of the VP8 protein. The antigenic domains 8-1, 8-2, 8-3, and 8-4 are represented by the colors green, purple, yellow, and blue, respectively. The conserved antigenic domain is shown in red. (F) The structure of the VP5 protein. The green represent the antigenic domains. The red represent the conserved antigenic domains. (G) The structure of the VP7 protein. The antigenic domains 7-1a, 7-1b, and 7-2 are represented by the colors green, blue and purple.

Structural modifications in antigenic epitopes of the VP7 trimer and VP4 multimer (composed of VP5* and VP8*) may compromise vaccine efficacy. As previously reported, proteolytic cleavage of the VP4 spike protein by trypsin generates two fragments: VP8* (26 kDa) and VP5* (60 kDa) (Aoki et al., 2009; Dormitzer et al., 2002). The VP8* subunit contains four surface-exposed antigenic epitopes (8-1 to 8-4), while the VP5* subunit harbors five antigenic regions (5-1 to 5-5) (Fig. 4). RVA strains, including the ZJ03 strain analyzed here, conserve critical trypsin cleavage sites at arginine residues (positions 231, 241, 247, 467, 582) and a lysine residue (position 258) (Miao et al., 2022) (data not shown).

Comparative analysis of ZJ03 VP7 neutralizing epitopes with reference strains revealed conserved residues in epitope 7-1 (98W, 104Q, 201Q) and epitope 7-2 (223E, 264G) (Fig. 4A). Sequence divergence was observed between ZJ03 and AHFY2022 at position 96 in both epitopes. Notably, SD-1 differed from AHFY2022 at three positions (96, 212, 221), while NJ2012 exhibited four divergent residues (96, 123, 212, 221) compared to ZJ03. Spatial mapping demonstrated heterogeneous distribution of VP7 epitope variations (Fig. 4G). Structural visualizations were generated using PyMOL (http://www.pymol.org).

In VP5* neutralizing epitopes, eight conserved residues were identified (306Y, 386G, 388Y, 393P, 398P, 429F, 440F, 459N) among porcine rotavirus (PoRV) strains (Fig. 4B). Strikingly, only one conserved position (100D in epitope 8-1) was observed across 25 analyzed VP8* epitope positions (Fig. 4C). Three-dimensional mapping revealed heterogeneous distribution of VP4 epitope variations on the molecular surface (Fig. 4E and 4F).

### 3.5 Pathogenicity in 3-day-old pig

**Fig. 5:**
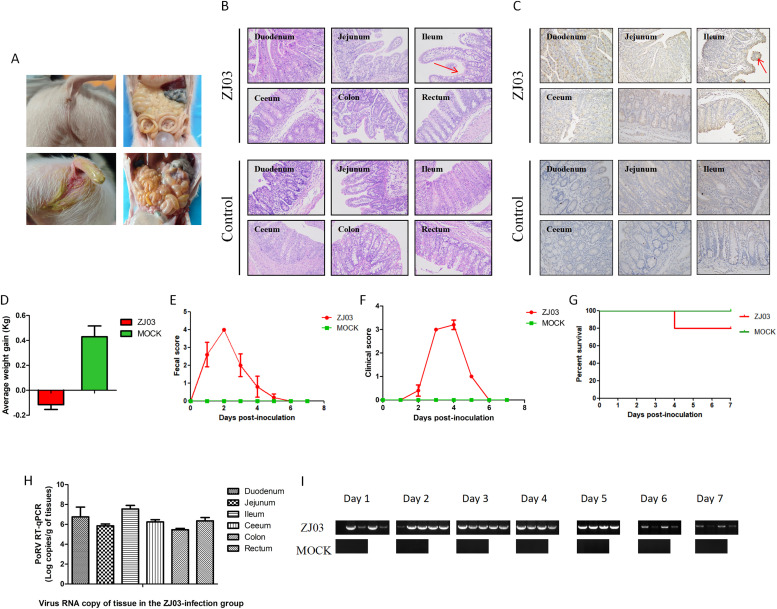
Pathogenicity of PoRV strain ZJ03 in 3-day-old piglets. 3-day-old piglets were orally inoculated with 2 mL ZJ03 virus and 2 mL DMEM (Control group) respectively. (A) The Clinical signs and macroscopic lesions. (B) HE results for histopathological lesions. (C) IHC results for histopathological lesions. (D) Average weight gain from days 0 to 7 post-challenged. (E) Fecal score was recorded each 24 h after inoculation until necropsy. (F) Clinical score was recorded each 24 h after inoculation until necropsy. (G) Survival curves of the piglets in both group. (H) Viral load in intestinal tissues was detected using RT-qPCR after necropsy. (I) Viral shedding was detected using RT-PCR each 24 h after inoculation until necropsy.

To evaluate the pathogenicity of the ZJ03 strain, five 3-day-old piglets were orally challenged with 2 mL per piglet of ZJ03 (F9) virus at a dose of 1 × 10^5.5 TCID_50_/mL, while three control piglets received DMEM. Necropsy at 7 dpi revealed characteristic gross lesions in challenged animals, including intestinal wall thinning, translucency, and luminal accumulation of yellowish fluid (Fig. 5A). Histopathological analysis demonstrated small intestinal villus atrophy, particularly in the ileum (Fig. 5B). Immunohistochemistry confirmed PoRV antigen localization within epithelial cells of atrophied ileal villi (Fig. 5C). Control piglets exhibited no macroscopic or microscopic abnormalities. All challenged piglets (ZJ03 group) developed marked dehydration and weight loss, whereas control animals remained clinically normal throughout the study (Fig. 5D). Diarrhea onset occurred in all challenged piglets by 24 h post-inoculation (hpi), with 3/5 exhibiting severe symptoms at this timepoint (Fig. 5E and 5F). All piglets occurred severe diarrhea at 48 hpi and persistent 1∼5 day, but the piglets gradually recovered thereafter (Fig. 5E and 5F), One mortality was recorded at 96 hpi (Fig. 5G), with peak clinical severity observed at 3-4 dpi (Fig. 5E and 5F). High viral RNA loads were identified throughout intestinal tissues of infected piglets, while control animals showed no detectable viral RNA (Fig. 5H). Fecal viral shedding was quantified using RT-PCR targeting the VP6 gene. PoRV RNA was detected in 4/5 rectal swabs at 24 hpi, with all fecal samples testing positive by 48 hpi until necropsy (Fig. 5I).

## 4 Discussion

This study aimed to isolate and characterize a novel rotavirus strain from diarrheic swine, elucidate its genomic characteristics and pathogenicity, identify the potential for interspecies transmission, and evaluate associated zoonotic risks. Fecal specimens were collected from swine exhibiting acute diarrhea and processed using standardized virological protocols for viral isolation. Following pretreatment and purification, isolates were propagated in MA104 cell cultures. The isolated strain (designated ZJ03) was characterized through electron microscopy and multicycle growth kinetics, confirming typical rotavirus morphology and replication dynamics. Viral RNA extracted from F9-passage cultures underwent high-throughput sequencing and de novo assembly. Comparative genomic analysis revealed ZJ03 as a G9P[23] strain exhibiting high sequence identity with cognate genotypes while harboring unique mutations in virulence and host adaptation genes. Pathogenicity was confirmed in specific pathogen-free piglets, with inoculated subjects developing severe clinical signs, sustained viral shedding, and significant histopathological alterations in intestinal tissues. Its high pathogenicity and unique genetic composition suggest a need for further investigation to assess public health risks, zoonotic potential, and implications for wildlife conservation, particularly regarding giant pandas.

This study characterizes the emergence and evolutionary origins of the novel porcine rotavirus strain ZJ03 (G9P[23]), a highly virulent variant in piglets. High-throughput sequencing and phylogenetic reconstruction revealed it’s a unique genomic constellation (G9-P[23]-I5-R1-C1-M1-A8-N1-T1-E1-H1). The VP3 gene exhibited 95.73 % nucleotide identity to a giant panda-derived RVA strain (HQ641295.1), while the NSP1 gene showed 94.64 % homology to the canine RVA strain RVA/Dog-tc/CHN/SCCD-A/2017/G9P[23] (MH910069.1). Notably, the canine strain SCCD-A itself has pig-related origins (Yan et al., 2019). Phylogenetic analysis the VP3 segment further demonstrated comparable genetic distances between ZJ03 and both the panda isolate (95.73 % identity) and porcine strain CN127 (94.23 % identity), precluding definitive assignment of its host origin. The remaining genomic segments clustered within established porcine RVA lineages. This porcine G9P[23] rotavirus with gene segments linked to canine and giant panda strains, exemplifies genetic plasticity in RVAs and underscores domestic swine as potential "mixing vessels" for interspecies reassortment. The incorporation of genetic material from wildlife (giant panda), companion animals (dog), and livestock (pig) highlights the dynamic evolutionary landscape of RVAs at the livestock-wildlife interface.

The porcine G9P[23] rotavirus, which contains gene segments linked to those of canine and giant panda strains, raises critical questions about cross-species transmission networks. This is particularly significant in areas where swine farming overlaps with wildlife habitats and domestic canine populations. The zoonotic implications of these findings are underscored by growing evidence that porcine RVAs contribute to human and canine infections through reassortment (Wandera et al., 2021; Akari et al., 2023;Yan et al., 2019). The increasing frequency of RVA recombination events, driven by co-circulation of diverse strains in multi-host ecosystems (Sadiq et al., 2018; Yahiro T, et al., 2018; Flores et al., 2021) further amplifies risks of virulent variant emergence. This evolutionary trajectory mirrors global trends where RVAs exploit ecological overlaps between species to evade host restrictions, necessitating integrated surveillance at the human-livestock-wildlife interface.

The G9 genotype of RVA represents a critical zoonotic threat, with phylogenetic evidence supporting shared ancestry between human and porcine strains in overlapping regions (Wang et al., 2024). Since its re-emergence as a dominant human genotype in the mid-1990s, G9 has concurrently risen in swine populations, now accounting for 11.4 % of Asian RVA infections (Wu et al., 2017). In China, while G5P[7] remains the predominant porcine genotype globally (Zhang et al., 2024; Qiao et al., 2024), epidemiological shifts highlight the increasing prevalence of G9P[23], G9P[7], and G9P[13] strains in diarrheic pigs (Monteagudo et al., 2022; Wu et al., 2017). This adaptive success underscores G9’s capacity to bridge host species barriers. Critically, the ZJ03 strain’s G9P[23] profile diverges from the G5P[7]/G5P[11] genotypes targeted by China’s commercial triple attenuated vaccine (Tang et al., 2024). This antigenic mismatch, combined with live-attenuated vaccines may reassort with field strains, explains the observed vaccine inefficacy and risks of viral diversification. Notably, ZJ03’s VP4 and VP7 antigenic epitopes harbor unique substitutions in surface-exposed residues (Fig. 4), including regions critical for neutralizing antibody binding. Comparative analysis revealed significant nucleotide and amino acid divergence between ZJ03 and other porcine G9P[23] strains, suggesting adaptive evolution to evade host immunity.

Comparative analysis of G9P[23] rotavirus pathogenicity reveals distinct virulence patterns across strains. Previous studies report that 3-day-old piglets infected with the HN03 (G9P[23]) strain developed severe diarrhea within 24 hpi, resolving by 72 hpi (Wang et al., 2018b), while PRG942 (G9P[23]) and PRG9121 (G9P[7]) infections induced diarrhea persisting for 1-8 dpi (Kim et al., 2013). Similarly, piglets challenged with the CN127 strain at 1 day old (4 ml/piglet, 1.0 × 10^7.0 TCID_50_/mL) experienced the onset of diarrhea between 6-24 h post-infection (hpi), with piglets euthanized at 48 hpi (Miao et al., 2022). In contrast, piglets challenged with the AHFY2022 strain at 5 days old (2 mL/piglet, 1.0 × 10^6.5 TCID_50_/mL) all developed diarrhea by 48 hpi, which resolved within the subsequent 24 h (Wang et al., 2024). In this study, experimental infection with the ZJ03 strain (2 mL/piglet, 1.0 × 10^5.5TCID_50_/mL) in 3-day-old piglets resulted in earlier symptom onset (diarrhea at 24 hpi), prolonged clinical severity (persisting through 7 dpi), and 100 % morbidity with one mortality by 96 hpi (Fig. 5). Necropsy revealed severe intestinal pathology, including luminal distension with yellowish fluid, gas accumulation, and ileal villous atrophy-a hallmark of rotavirus enteritis. Immunohistochemistry confirmed high viral antigen loads localized to atrophic ileal villi (Fig. 5C), correlating with sustained fecal viral shedding throughout the study period. Control piglets showed no clinical signs, viral shedding, or intestinal pathology, confirming ZJ03-specific pathogenicity. These pathogenic features correlate with ZJ03’s efficient in vitro replication (peak titer 10^9.25 TCID_50_/mL at 36 hpi), indicating adaptive mutations that warrant further investigation. Antigenic analysis of VP7 and VP4 epitopes revealed unique substitutions in ZJ03, particularly in regions critical for neutralizing antibody recognition. This divergence may explain its evasion of vaccine-induced immunity and underscores the challenges in developing broadly protective vaccines.

The detection of a porcine G9P[23] RVA strain, with gene segments linked to canine and giant panda strains, carries significant One Health implications. These findings underscore two urgent priorities: (1) development of updated vaccines incorporating contemporary genotypes like G9P[23], and (2) enhanced surveillance to detect antigenic drift and interspecies reassortment. The latter is particularly vital given ZJ03’s hybrid genome, which incorporates genes from giant panda and canine RVAs-a hallmark of swine’s role as "mixing vessels" in regions where livestock, wildlife, and domestic animals intersect. Proactive biosecurity measures and multidisciplinary One Health strategies are essential to mitigate zoonotic spillover and curb the emergence of virulent reassortants.

## CRediT authorship contribution statement

**Xi Li:** Writing – review & editing, Writing – original draft, Software, Methodology, Investigation, Formal analysis, Data curation. **Jingjing Wang:** Resources, Methodology, Data curation. **Yuankui Zhang:** Methodology, Formal analysis, Data curation. **Yarong Zhao:** Writing – review & editing. **Wenjun Liu:** Writing – review & editing, Formal analysis, Data curation. **Yanli Shi:** Writing – review & editing, Validation, Supervision, Conceptualization.

## Declaration of competing interest

The authors affirm that none of the work described in this manuscript could have been influenced by any conflicting financial interests or personal relationships they may have. All authors have approved the manuscript for publication. On behalf of my coauthors, I would like to state that the work described was original research that has never been published before and is not currently being considered for partial or full publication anywhere. The enclosed manuscript has the approval of all listed authors.

## Data Availability

Data will be made available on request.
